# Development and Evaluation of Digital Game-Based Training for Managers to Promote Employee Mental Health and Reduce Mental Illness Stigma at Work: Quasi-Experimental Study of Program Effectiveness

**DOI:** 10.2196/mental.7600

**Published:** 2017-08-04

**Authors:** Sabine Elisabeth Hanisch, Ulrich Walter Birner, Cornelia Oberhauser, Dennis Nowak, Carla Sabariego

**Affiliations:** ^1^ Department of Medical Informatics, Biometry and Epidemiology Chair for Public Health and Health Services Research, Research Unit for Biopsychosocial Health Ludwig-Maximilians-University Munich Munich Germany; ^2^ HR EHS Department of Psychosocial Health and Well-being Siemens AG Munich Germany; ^3^ Department of Medical Informatics, Biometry and Epidemiology-IBE Chair for Public Health and Health Services Research, Research Unit for Biopsychosocial Health Ludwig-Maximilians-University Munich Munich Germany; ^4^ Institute and Outpatient Clinic for Occupational, Social and Environmental Medicine WHO Collaborating Centre for Occupational Health Clinic of Ludwig-Maximilians-University Munich Munich Germany

**Keywords:** stigma, mental health, workplace, prevention, health promotion, leadership, eHealth, Internet

## Abstract

**Background:**

To counteract the negative impact of mental health problems on business, organizations are increasingly investing in mental health intervention measures. However, those services are often underused, which, to a great extent, can be attributed to fear of stigmatization. Nevertheless, so far only a few workplace interventions have specifically targeted stigma, and evidence on their effectiveness is limited.

**Objective:**

The objective of this study was to develop and evaluate a digital game-based training program for managers to promote employee mental health and reduce mental illness stigma at work.

**Methods:**

We describe the empirical development of Leadership Training in Mental Health Promotion (LMHP), a digital game-based training program for leaders. A 1-group pre-post design and a 3-month follow-up were used for training evaluation. We applied multilevel growth models to investigate change over time in the dependent variables knowledge, attitudes, self-efficacy, and intentions to promote employee mental health in 48 managers of a global enterprise in the United Kingdom. Participants were mainly male (44/48, 92%) and ranged in age from 32 to 58 (mean 46.0, SD 7.2) years.

**Results:**

We found a positive impact of the Web-based training program on managers’ knowledge of mental health and mental illness (*P*<.001), on attitudes toward people with mental health problems (*P*<.01), and on their self-efficacy to deal with mental health situations at work (*P*<.001), with the exception of intentions to promote employee mental health, which was initially high.

**Conclusions:**

Results provide first evidence of the effectiveness of LMHP to positively affect managers’ skills to promote employee mental health at work. Furthermore, the high rate of participation in LMHP (48/54, 89%) supports the use of digital game-based interventions to increase user engagement and user experience in mental health programs at work.

## Introduction

Due to their high prevalence (1 in 4) [1], the economic impact of mental health problems such as depression can be considerable for employers globally. In high-income countries, the trend of sick days lost due to mental health problems has been growing in recent years [2]. Resulting total work loss due to sickness absence, lost at-work productivity, and turnover are estimated to cost £26 billion a year in the United Kingdom alone [3].

To counteract the negative impact of mental health problems on business, organizations are increasingly investing in mental health promotion, prevention, and intervention efforts [4]. For example, many organizations have implemented employee assistance programs (EAPs), which typically offer assessment, counselling, and referral services to employees with work-related or personal problems [5]. Others offer stress reduction programs such as meditation or relaxation interventions [6].

However, there are a few drawbacks worth discussing with regard to the current practice of workplace mental health promotion. First, most interventions aiming to promote employee mental health focus on the employee level (such as in stress management) while neglecting the organizational level (working conditions) [7,8]. However, many factors that positively affect employee mental health are related to the social environment at work, such as the working culture, level of social support, and leadership style [9]. Second, with regard to leadership, few efforts have considered the special role of managers in organizations [10,11]. Because they are in close contact with employees, managers are in the best position to spot signs of deteriorating mental health early and to provide support. Unfortunately, however, many leaders lack training in the management of workplace mental health and thus are ill-equipped to support affected individuals adequately [12]. Third, utilization rates of EAPs are generally low [5,13]. Despite the availability of effective mental health interventions, the majority of affected individuals choose not to seek help [14]. A major barrier that strongly contributes to the underuse of EAPs is the stigma associated with mental illness [15,16].

Stigma is defined as (1) the lack of knowledge of mental health problems and treatments, (2) prejudicial attitudes, and (3) the lack of supportive behavior, or anticipated or real acts of discrimination against people with mental health problems [14]. Despite its far-reaching impact on employees’ willingness to seek help, current practices in mental health promotion largely fail to address stigma specifically [17]. Therefore, as of yet, efforts in mental health promotion do not seem to reach their primary aim, early and effective treatment, satisfactorily [18]. Instead, raising awareness of the importance of mental health, reducing stigma, and creating an organizational culture of acceptance, diversity, and respect may be a necessary prerequisite for the acceptance, use, and, thus, effectiveness of mental health interventions such as EAPs [19].

While the majority of stigma reduction programs targeted the general population—for example, in public health campaigns—there is growing interest in the effectiveness of workplace antistigma interventions [20,21]. A systematic review [17] found that workplace antistigma interventions can have a positive impact on employees’ knowledge, attitudes, and supportive behavior toward people with mental health problems. However, limitations became apparent: (1) most interventions targeted the public sector, (2) half of the studies included did not target all 3 dimensions of stigma, which is key in achieving ultimate behavioral change, (3) there is a lack of evidence concerning the sustainability of workplace antistigma interventions due to insufficient follow-up beyond pre- and postintervention assessments, and (4) most interventions were delivered face-to-face, thus having only a limited reach and impact on stigma among the wider workforce.

The dissemination of digital interventions, however, could be a powerful strategy to facilitate widespread behavioral and cultural change in organizations [22]. Compared with face-to-face interventions, digital interventions have many advantages, such as greater reach, reduced barriers to access, increased participant engagement and adherence to treatment, and flexible and self-paced learning, as well as being more cost effective [23]. However, most digital health promotion efforts so far have targeted physical rather than mental health and mainly focused on the treatment of specific disorders in a subgroup (eg, depression in teenagers) [24-26]. Evidence on digital interventions aiming to prevent mental health problems is still scarce and even more so with regard to the workplace setting [27,28]. This study, therefore, aimed to address some of the limitations of current practices in mental health promotion and of research on stigma reduction. We followed 2 objectives: (1) to develop a digital game-based intervention to train leaders of a private sector organization to effectively manage employee mental health by addressing all 3 dimensions of stigma in order to prevent mental health problems and promote an open, inclusive, and supportive working culture, and (2) to evaluate the intervention in terms of its effectiveness and mid-term sustainability in a pilot study.

Specifically, we hypothesized that our digital game-based intervention, called Leadership Training in Mental Health Promotion (LMHP), would lead to (1) improved mental health knowledge, (2) increased positive attitudes toward people with mental health problems, (3) increased self-efficacy to deal with mental health situations at work, and (4) improved intentions to promote employee mental health at work in managers undertaking the training.

## Methods

### Objective 1: Intervention Development

The intervention was developed in a collaborative effort between the department of psychosocial health and well-being of a large global private sector company, which employed around 348,000 employees in more than 100 countries in 2015, and the Chair for Public Health and Health Services Research of Ludwig-Maximilians-University (LMU) in Munich, Germany.

#### Approach

In developing LMHP, we followed a systematic approach similar to intervention mapping [29] for designing theory- and evidence-based health promotion programs. Specifically, we took several steps, from analyzing the problem of mental illness stigmatization and effective change methods [17], to assessing the needs for managerial training on mental health, and, finally, to developing the training, as well as an implementation and evaluation plan.

#### Content

We developed training content based on a review of workplace training programs on mental health [30-33] and on consultations with subject matter experts in the field of health management, human resources, and training and development. Furthermore, we carried out a needs assessment via 14 semistructured interviews (7 managers, 7 employees) in the participating organization, investigating managerial training needs in terms of preferred content and mode of delivery (unpublished data). Results indicated a particular need for managers to be trained in spotting warning signs of mental distress, and in how to interact with and support affected employees.

#### Format

While e-learning is well established in larger enterprises, Web-based training in its most common form, animated slidecasts, is losing more and more in attractiveness and acceptance [34]. To counteract low participant engagement [35], LMHP was developed as a simulation game, a Web-based training program combining elements of both games and simulations [36]. By creating a real in-person environment with all the complexities of the formal and particularly social interactions typically found in the workplace, the program provides managers with the opportunity to directly apply what they learned about people management and to practice new skills in a safe virtual environment [37]. This way, managers can get a sense of the potential impact of different leadership styles on employee mental health without having to worry about real-world consequences.

#### Gamification

To facilitate an innovative and engaging learning experience [35], we used a subtle form of gamification in LMHP to fit the sensitivity of the training content. Gamification is defined as “the use of game design elements in non-game contexts” [38]. For example, while we refrained from providing badges for achievements or enabling competition between players, we did include several gamification strategies that were found to increase engagement and learning [39]. Those involved providing a storyline and clear goals, including the capacity to overcome challenges by learning; providing feedback on performance; showing progress (in terms of how leader behavior affects employee mental health over time); and reinforcing learning by allocating points (eg, for quiz questions answered correctly).

### Objective 2: Intervention Evaluation

The goal of this pilot study was to evaluate the effectiveness of a digital game-based training program for managers, which we developed to promote employee mental health and reduce mental health-related stigma at work, using a 1-group pre-post design and a 3-month follow-up. The pilot study was carried out at a defined site of the participating organization near Oxford, United Kingdom.

#### Participants

All managers of this site were invited to take part in LMHP and its associated research study. To be included, participants had to be of working age (between 18 and 65 years) and be managing at least one employee at the time of the training. Informed consent was obtained from all individual participants included in the study.

#### Procedure

Invitations to participate in LMHP were sent out by email approximately a week in advance of the scheduled Web-based training. This invitation notified participants about the study’s objectives, potential risks, data protection, etc.

Participants were then sent a personal link that allowed (1) participants to give their informed consent to participate in this study, (2) participants to access the training program for a limited time period of 3 weeks, (3) participants to access the pre- and postquestionnaire immediately before (T1) and after (T2) completion of the training, and (4) the researchers to allocate responses at T1, T2, and T3 to an individual. However, the link did not include any information that could be used to identify participants. At T3 (12 weeks after training completion), participants were resent their personal link in order to fill in a follow-up questionnaire to evaluate the first mid-term effects of the intervention.

Any communication about the training initiative (eg, invitations), as well as personal links to training and questionnaires, was sent out via email by a human resources staff member of the participating organization, who was not involved in the study. Questionnaires were completed anonymously online, and responses were tracked and stored safely at the external training provider. The external training provider then replaced participants’ email addresses with a random, unique 3-digit identifier and posted the data back to the researchers at LMU Munich. To increase response rates, the external training provider informed the human resources staff member of the participating organization about any nonresponders so that he could send out reminders. The researchers were never told the names of individual respondents, and the human resources staff member in the participating organization never saw any completed questionnaires or individually identifiable data.

#### Ethics

Ethical approval for the study was given by the Ethics Committee of LMU Munich, Germany. All procedures performed in studies involving human participants were in accordance with the ethical standards of the institutional or national research committee and with the 1964 Declaration of Helsinki and its later amendments or comparable ethical standards.

#### Outcome Measures

Demographic questions included age, sex, level of education, marital status, whether they currently lived alone, and whether they knew someone with a mental health problem and had been diagnosed with or treated for a mental health problem themselves.

Other outcome measures matched the knowledge, attitudinal, and behavioral dimensions of stigma as defined above. We administered 4 validated instruments. To all of them, a 5-point Likert scale ranging from 1 (“strongly disagree”) to 5 (“strongly agree”) was applied. We calculated global scores on all instruments using sum scores, with higher scores indicating a better outcome, with the exception of stigmatizing attitudes. All measures were administrated at all 3 time points.

#### Knowledge

We assessed knowledge about mental health problems using the first 6 items, which are related to stigma, of the 12-item Mental Health Knowledge Schedule (MAKS) [40]. An example item is “Psychotherapy can be an effective treatment for people with mental health problems.” Sum scores ranged from 6 to 30.

Additionally, we developed a set of 7 quiz questions to test participants’ knowledge on specific training content of LMHP, with 3 answer options, of which 1 was correct. An example item is “Which statement about business costs related to mental disorders is correct?” In this case, sum scores ranged from 0 to 7.

#### Attitudes

We assessed attitudes in the workplace toward coworkers who may have a mental illness using the 23-item Opening Minds Scale for Workplace Attitudes (OMS-WA), an adapted version of the Opening Minds Scale for Health Care Providers (OMS-HC) [41]. OMS-WA consists of 5 subscales: 6 items on avoidance, 5 on perceived dangerousness, 5 on work beliefs and competencies, 4 on helping, and 3 on responsibility of people with mental health problems. During evaluation, we considered attitudes as a whole, with sum scores ranging from 23 to 115, as well as the individual subscales, with sum scores ranging from 6 to 30 for avoidance, 5 to 25 for perceived dangerousness, 5 to 25 for work beliefs and competencies, 4 to 20 for helping, and 3 to 15 for responsibility. An example item is “I would try to avoid a coworker with a mental illness.”

#### Behavior

To assess behavioral change in leaders, we used proxy variables (eg, self-efficacy to deal with mental health situations at work and intentions to promote employee mental health), since in a 3-month period not very many mental health situations are likely to arise at work where leaders could possibly demonstrate actual support. However, prior research found that enhanced intentions and high self-efficacy increase the likelihood that a person will engage in newly learned behaviors [42].

In this study, we measured self-efficacy with regard to managing employee mental health by a previously adapted version of the 9-item New General Self-Efficacy Scale [30,43]. Items included “When facing difficulties related to employee mental health, I am certain that I will handle them appropriately.” Sum scores ranged from 9 to 45.

To assess participants’ intentions to promote employee mental health, we used a previously adapted 3-item version of a safety scale designed to assess managers’ safety promotion intentions [30,44]. An example item is “I want to apply what I learn about employee mental health to my work setting.” Sum scores ranged from 3 to 15.

#### Statistical Methods

We used descriptive statistics (mean, median, SD) to describe the study population. Multilevel growth models (with random intercept) were applied to investigate change over time in the dependent variables knowledge, attitudes, self-efficacy, and intentions to promote employee mental health [45]. An advantage of multilevel growth models is that missing data can be handled flexibly (using likelihood-based estimation) and thus allowed incorporation of all available data. First, we used time as a fixed factor in the models, as pre- and postmeasurements were collected on the same day for each participant and variability in time from post- to follow-up measurements was very low across participants. Second, we investigated whether selected participant characteristics (age, educational level) predicted initial status. We applied the forward modelling approach, starting with models without any predictors (model A) and adding potential explanatory variables as fixed effects at subsequent steps (models B and C). To select the best model, we considered reductions of deviance (–2*log likelihood) and of Akaike information criterion and Bayesian information criterion values, with smaller values indicating a better-fitting model. We computed change as the difference in relation to the baseline (T1) score. Parameter estimates and standard errors (SE) are reported. Effects were judged significant at alpha≤.05, unless otherwise noted. Statistical analyses were performed using IBM SPSS 23.0 and SPSS MIXED (IBM Corporation).

## Results

### Objective 1: Intervention Development

Taking all formative research described above into consideration, we designed LMHP in a way to train managers in (1) understanding mental health and mental illness, (2) spotting warning signs, (3) taking early and appropriate action, and (4) monitoring and self-monitoring.

#### Digital Game-Based Learning

The training consisted of one single session, which took between 1.5 and 2 hours to complete, thereby meeting managers’ expectations of a particularly concise and time-efficient training format as expressed during interviews (see formative research described above). The setting was the office hub where, over a virtual time period of 7 weeks, the player was put into the position of a manager. During that time period, it was the manager’s task to supervise a virtual team and manage employee mental health effectively.

**Table 1:** Outline of content and psychological constructs covered in the virtual scenarios of the Leadership Training in Mental Health Promotion program.

Scenario		Objective	Knowledge	Attitude	Skills
1.	Psychological well-being	Promotion of mental health	Create awareness of the importance of mental health at work and that stress or mental illness affects everyone	Develop more positive attitudes toward promoting mental health at work	Communication and behavioral strategies to ensure that healthy employees stay healthy
2.	Acute stress	Prevention of mental illness	Create awareness that acute stress can result in psychological as well as physical symptoms	Develop more positive attitudes toward discussing the topic of stress more openly at work and to promote employee mental health	Communication, identification of warning signs, support strategies
3.	Chronic stress	Prevention of mental illness	Create awareness that persistent stress has severe detrimental effects on the body and the mind and, if not dealt with, can lead to long-term sickness absence	Develop more positive attitudes toward employees with mental health problems with regard to avoidance, work competency, responsibility, and helping	Communication, identification of warning signs, and support and referral strategies
4.	Mental Illness	Rehabilitation and return to work	Create awareness of common mental health problems and of return-to-work policies and procedures	Develop more positive attitudes toward employees with mental health problems with regard to perceived dangerousness, work competency, responsibility, avoidance, and helping	Communication, planning a successful return to work, workplace accommodations, monitoring, actively counteracting stigma and discrimination, facilitating open discussions

The virtual team consisted of 4 employees showing diverse psychological profiles; thus, each represented a different mental health scenario likely to appear in real office life. Scenarios contained examples of the promotion of mental health, the prevention of mental illness, and the rehabilitation of employees with common mental health problems such as anxiety or depressive disorders (see Table 1). Due to their relatively low prevalence rates, more severe mental disorders such as psychosis were not addressed in this workplace training. All scenarios required managers to develop and practice their skills in spotting warning signs, taking (early) action, and monitoring employees while building knowledge of mental health and mental illness and more positive attitudes toward employees with mental health problems at the same time (see Table 1).

For example, to sensitize managers in the recognition and identification of warning signs, certain hints were placed into the virtual work environment (eg, medication, uneaten lunch, or work piling up on an employee’s desk) that may or may not signal a growing underlying mental imbalance. Once the manager had spotted something unusual or alarming, he or she could choose to engage in a conversation with the respective employee. Different dialogue options were provided to choose from, which were more or less appropriate given the sensitivity of a certain topic. Depending on how the manager behaved, the respective employee chose to either shut down and end the conversation or open up and share further information the manager needed to be able to offer appropriate and effective support.

To ensure continuous learning and improved self-efficacy to manage mental health situations at work, the player was provided with instant feedback regarding his or her actions after the end of each conversation. Furthermore, a video of an actual affected employee of the participating organization sharing his or her experience with burnout was shown automatically to every player. The personal testimonial was presented in a way to counter prominent stereotypes of people with mental health problems and with a strong focus on the road toward recovery and well-being, thus involving many features considered fundamental to reducing stigma [46]. This video formed a very powerful part of the training, since contact with people with lived experience (face-to-face or video-based) is argued to be the strongest method to tackle mental illness stigma [47].

#### Mental Health Toolbox

Next to scenario-based learning, LMHP also offered a mental health toolbox that provided managers with practical information on topics found to be relevant to manage a given scenario successfully. The toolbox was presented in a way to improve managers’ knowledge of mental health and mental illness, improve their attitudes toward employees with mental health problems, and train them in skills to deal with mental health situations at work effectively. Topics of the mental health toolbox focused on 4 main areas: what mental health and mental illness mean, how to recognize signs of mental distress, how to start a conversation, and how to support affected employees effectively (see Table 2). Furthermore, the toolbox aimed to facilitate the application of newly learned skills in real everyday office life. For example, checklists with warning signs or guidelines for conversations on mental health could be downloaded as pdf files and serve as useful aids in interactions with employees.

**Table 2:** Outline of content and psychological constructs covered in the Mental Health Toolbox of the Leadership Training in Mental Health Promotion program.

Focus areas of training		Module	
A	Understanding mental health and mental illness	A1	Mental health affects us all
		A2	Understanding mental health and mental illness
		A3	Economic impact of mental illness
		A4	Risk factors and treatment of mental disorders
B	Recognizing signs of mental distress	B1	What is stress?
		B2	Work-related stressors and resources
		B3	Warning signs
		B4	Common mental disorders at work
C	Starting the conversation	C1	Stigma: a barrier to help-seeking
		C2	Communication techniques
		C3	Guidance for leaders
		C4	In-house support services
D	Supporting effectively	D1	Key role of managers
		D2	Providing support
		D3	Return to work
		D4	Self-care

#### Theoretical Foundation and Underlying Models

The idea behind the training—for example, the progression of employees’ mental state in scenarios—followed the principles of the mental health continuum model [48,49]. This model postulates that mental health is spread out along a continuum, meaning that people are not either mentally healthy or mentally ill, but that they can move in and out of further phases in between.

In LMHP, we used an adapted version of the mental health continuum model to suit our specific needs. Each phase of this continuum (health, acute stress, chronic stress, and illness) is assigned certain warning signs and recommended actions to take as an affected individual but also as a manager supporting affected employees. In this way, mental health becomes more concrete, which, in turn, facilitates managers’ understanding of mental health and warning signs.

On several occasions during the training, the manager was asked to assess each employee’s mental state along the phases of the mental health continuum model. Afterward, the player was given feedback on an employee’s actual mental state and on other parameters the manager influenced with his or her behavior, such as perceived managerial support or an employee’s willingness to seek professional help. This exercise was designed to improve managers’ self-efficacy in identifying warning signs and to strengthen their intentions to promote employee mental health.

### Objective 2: Intervention Evaluation

#### Participants

**Figure 1:**
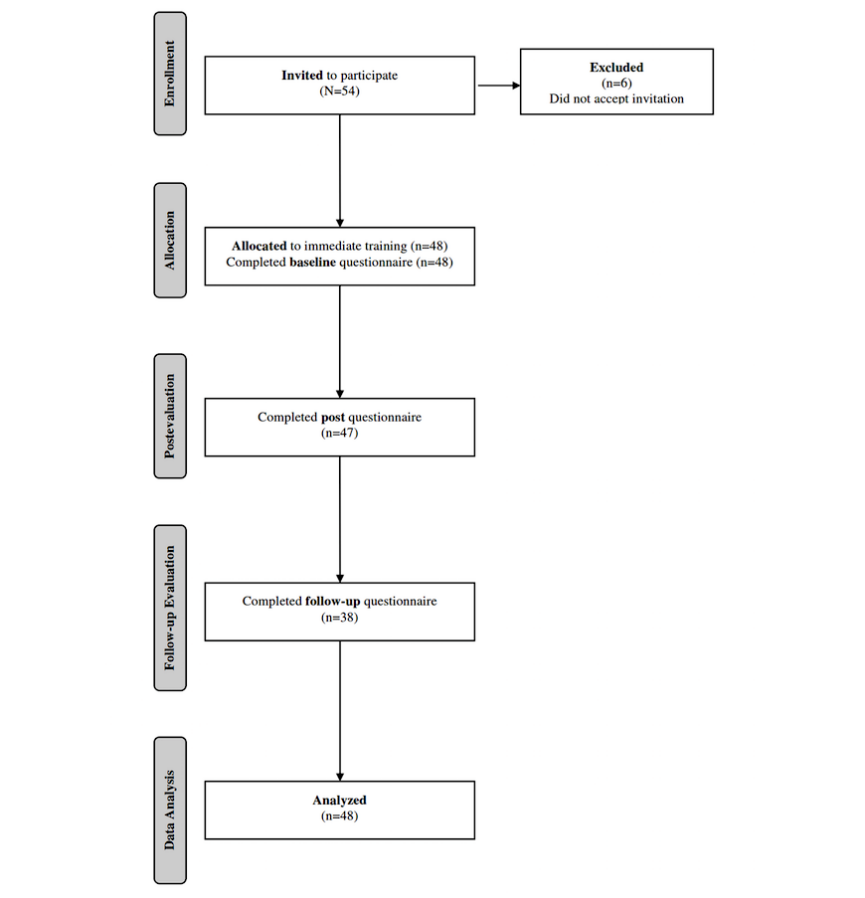
Flow diagram showing progress through the phases of the trial.

Figure 1 shows the flow of participants at each stage of the study. Of 54 managers working at the site, 48 (89%) accepted our invitation, completed the baseline questionnaire, and took part in the training. Of the 48 participants, 47 (98%) completed the postquestionnaire immediately after the training and 38 (79%) responded to the follow-up questionnaire 3 months later. Complete data from 3 waves were available for 37 (77%) participants and from at least two waves for 47 (98%) respondents.

#### Descriptive Analysis

Table 3 presents baseline demographic characteristics of the sample population: 92% of participants were male (44/48). Participants ranged in age from 32 to 58 (mean 46.0, SD 7.2) years. Among the 48 participants, 48% (23/48) had a university degree, 77% (37/48) were married, and 88% (42/48) were not living alone. Furthermore, 63% (30/48) knew someone with a mental health problem and 10% (5/48) had been diagnosed with or treated for a mental health problem themselves. Finally, 17% (8/48) received further training on mental health between the postevaluation and follow-up evaluation.

**Table 3:** Baseline demographic characteristics of the sample population (n=48).

Characteristics		Data
Age in years, mean (SD), median		46.0 (7.2), 45.5
**Age groups^a^, n (%)**		
	<45.5 years	24 (50)
	≥45.5 years	24 (50)
**Sex, n (%)**		
	Male	44 (92)
	Female	4 (8)
**Education level attained, n (%)**		
	Graduate degree	11 (23)
	Bachelor’s degree	12 (25)
	Nonuniversity certificate	13 (27)
	High school	10 (21)
	Less than high school	2 (4)
**Education groups^a^, n (%)**		
	University degree	23 (48)
	Nonuniversity degree	25 (52)
**Marital status**		
	Married	37 (77)
	Divorced or separated	6 (13)
	Single	3 (6)
	Common-law relationship	2 (4)
**Living alone, n (%)**		
	No	42 (88)
	Yes	5 (10)
	Prefer not to answer	1 (2)
**Know someone with mental health problem, n (%)**		
	No	13 (27)
	Yes	30 (63)
	Prefer not to answer	5 (10)
**Been diagnosed with or treated for mental health problem, n (%)**		
	No	41 (85)
	Yes	5 (10)
	Prefer not to answer	2 (4)
**Received further training postintervention, n (%)**		
	No	30 (63)
	Yes	8 (17)
	Missing values	10 (21)

^a^Variables included in multilevel analysis (model C).

**Table 4:** Descriptive statistics for respondents who participated at all 3 time points^a^ (n=37).

Measures	Wave 0		Wave 1		Wave 2	
	Mean	SD	Mean	SD	Mean	SD
Knowledge (MAKS^b^)	22.1	2.6	24.2	2.5	24.0	2.8
Knowledge (quiz)	4.4	1.4	5.6	1.4	4.9	1.2
Attitude total	45.9	10.7	43.1	11.5	42.3	10.3
Attitude avoidance	11.4	3.6	10.1	3.0	9.8	3.2
Attitude dangerousness	10.5	3.0	9.3	3.3	9.1	2.7
Attitude work	10.9	3.0	11.2	3.3	10.4	3.1
Attitude help	8.0	1.6	8.0	2.2	8.6	2.7
Attitude responsibility	5.0	2.0	4.5	1.6	4.4	1.7
Self-efficacy	31.5	3.6	34.7	3.4	34.2	2.9
Promotion intentions	12.2	1.3	12.4	1.2	12.3	1.2

^a^Wave 0, baseline; wave 1, postintervention; wave 2, 3-month follow-up.

^b^MAKS: Mental Health Knowledge Schedule.

#### Multilevel Analysis

Table 4 shows the mean scores of knowledge, attitudes, self-efficacy, and intentions to promote employee mental health at the 3 time points. In general, observed baseline scores indicated that, before the intervention, managers had quite good knowledge of mental health, fairly positive attitudes toward people with mental illness, and a high level of self-efficacy, as well as intentions to promote employee mental health.

Table 5 and Table 6 show the results of the multilevel analysis. Adding age and education (refer to Table 3) to the models neither showed significant effects regarding initial status nor improved the goodness of fit. Thus, in the following, we focused on results of model A intercept and, particularly, model B intercept and time. Overall, the B models had good fit. These models indicated that knowledge of mental health and mental illness (measured by MAKS and the quiz) and self-efficacy to deal with mental health situations at work significantly increased over time and that this effect remained significant over the 3-month period (see Table 5). Regarding stigmatizing attitudes, attitudes (total scale; Table 5) and attitude subscales related to avoidance, perceived dangerousness, and responsibility (Table 6) significantly decreased over time with these effects also being sustained 3 months later. However, attitudes related to work and competency beliefs and to helping people with mental health problems did not change over time (Table 6). Moreover, managers’ intentions to promote employee mental health did not change over time (Table 5).

**Table 5:** Mixed models (with random intercept) considering knowledge assessed by MAKS^a^, knowledge assessed by quiz, attitude (total), self-efficacy, and intentions to promote employee mental health as the dependent variable (n=48).

Dependent variable and predictors of change over time		Model A: unconditional means model		Model B: unconditional growth (with time)		Model C: time & age & education	
		Parameter estimate (SE)	*P* value	Parameter estimate (SE)	*P* value	Parameter estimate (SE)	*P* value
**Knowledge (MAKS)**							
	Fixed effects						
	Intercept (initial status)	23.27 (0.324)	<.001	21.98 (0.372)	<.001	21.84 (0.572)	<.001
	Time (rate of change)						
	Wave = 1			2.16 (0.335)	<.001	2.16 (0.335)	<.001
	Wave = 2			1.88 (0.361)	<.001	1.87 (0.361)	<.001
	Age					–0.09 (0.641)	
	Education					0.38 (0.642)	
	Variance components						
	Level 1: within-person (residual)	4.13 (0.633)	<.001	2.65 (0.407)	<.001	2.65 (0.407)	<.001
	Level 2: in intercept	3.51 (1.052)	.001	3.99 (1.024)	<.001	3.95 (1.017)	<.001
	Goodness of fit						
	Deviance	623.88		585.60		585.23	
	AIC^b^	629.88		595.60		599.23	
	BIC^c^	638.55		610.05		619.47	
**Knowledge (quiz)**							
	Fixed effects						
	Intercept (initial status)	5.01 (0.138)	<.001	4.38 (0.191)	<.001	4.36 (0.259)	<.001
	Time (rate of change)						
	Wave = 1			1.36 (0.239)	<.001	1.36 (0.239)	<.001
	Wave = 2			0.55 (0.256)	.03	0.53 (0.256)	.04
	Age					–0.34 (0.263)	
	Education					0.38 (0.642)	
	Variance components						
	Level 1: within-person (residual)	1.86 (0.284)	<.001	1.36 (0.208)	<.001	1.36 (0.208)	<.001
	Level 2: in intercept	0.24 (0.211)		0.40 (0.197)	.04	0.33 (0.185)	
	Goodness of fit						
	Deviance	474.48		446.59		443.09	
	AIC	480.48		456.59		457.09	
	BIC	489.15		471.04		477.32	
**Attitude (total)**							
	Fixed effects						
	Intercept (initial status)	43.77 (1.511)	<.001	46.13 (1.633)	<.001	47.93 (2.601)	<.001
	Time (rate of change)						
	Wave = 1			–3.49 (1.095)	.002	–3.49 (1.095)	.002
	Wave = 2			–4.08 (1.185)	.001	–4.06 (1.185)	.001
	Age					–1.09 (3.002)	
	Education					–2.64 (3.004)	
	Variance components						
	Level 1: within-person (residual)	33.47 (5.147)	<.001	28.33 (4.356)	<.001	28.34 (4.361)	<.001
	Level 2: in intercept	97.211 (22.562)	<.001	99.63 (22.644)	<.001	97.43 (22.218)	<.001
	Goodness of fit						
	Deviance	949.58		935.62		934.70	
	AIC	955.58		945.62		948.70	
	BIC	964.26		960.07		968.93	
**Self-efficacy**							
	Fixed effects						
	Intercept (initial status)	33.59 (0.396)	<.001	31.54 (0.507)	<.001	31.14 (0.742)	<.001
	Time (rate of change)						
	Wave = 1			3.62 (0.551)	<.001	3.62 (0.551)	<.001
	Wave = 2			2.78 (0.225)	<.001	2.77 (0.592)	<.001
	Age					0.47 (0.801)	
	Education					0.36 (0.801)	
	Variance components						
	Level 1: within-person (residual)	11.28 (1.752)	<.001	7.18 (1.113)	<.001	7.20 (1.119)	<.001
	Level 2: in intercept	3.41 (1.714)	.046	5.16 (1.685)	.002	5.03 (1.670)	.003
	Goodness of fit						
	Deviance	728.85		691.95		691.39	
	AIC	734.86		701.95		705.39	
	BIC	743.53		716.40		725.62	
**Promotion intentions**							
	Fixed effects						
	Intercept (initial status)	12.46 (0.151)	<.001	12.31 (0.185)	<.001	12.08 (0.269)	<.001
	Time (rate of change)						
	Wave = 1			0.36 (0.192)		0.36 (0.192)	
	Wave = 2			0.08 (0.207)		0.07 (0.207)	
	Age					0.00 (0.292)	
	Education					0.48 (0.292)	
	Variance components						
	Level 1: within-person (residual)	0.91 (0.140)	<.001	0.87 (0.135)	<.001	0.88 (0.136)	<.001
	Level 2: in intercept	0.76 (0.233)	.001	0.76 (0.231)	.001	0.70 (0.220)	.001
	Goodness of fit						
	Deviance	421.88		418.22		415.58	
	AIC	427.88		428.22		429.58	
	BIC	436.55		442.67		449.81	

^a^MAKS: Mental Health Knowledge Schedule.

^b^AIC: Akaike information criterion.

^c^BIC: Bayesian information criterion.

**Table 6:** Mixed models (with random intercept) considering attitudes regarding avoidance, dangerousness, workability, helping, and responsibility as the dependent variable (n=48).

Dependent variable and predictors of change over time		Model A: unconditional means model		Model B: unconditional growth (with time)		Model C: time & age & education	
		Parameter estimate (SE)	*P* value	Parameter estimate (SE)	*P* value	Parameter estimate (SE)	*P* value
**Attitude avoidance**							
	Fixed effects						
	Intercept (initial status)	10.50 (0.439)	<.001	11.44 (0.492)	<.001	11.69 (0.773)	<.001
	Time (rate of change)						
	Wave = 1			–1.37 (0.390)	.001	–1.37 (0.390)	.001
	Wave = 2			–1.66 (0.422)	<.001	–1.66 (0.422)	<.001
	Age					–0.39 (0.880)	
	Education					–0.12 (0.881)	
	Variance components						
	Level 1: within-person (residual)	4.43 (0.681)	<.001	3.60 (0.554)	<.001	3.60 (0.555)	<.001
	Level 2: in intercept	7.63 (1.926)	<.001	8.00 (1.932)	<.001	7.95 (1.924)	<.001
	Goodness of fit						
	Deviance	659.03		641.77		641.55	
	AIC^a^	665.03		651.77		655.55	
	BIC^b^	673.70		666.22		675.78	
**Attitude dangerousness**							
	Fixed effects						
	Intercept (initial status)	9.72 (0.404)	<.001	10.60 (0.440)	<.001	11.33 (0.688)	<.001
	Time (rate of change)						
	Wave = 1			–1.32 (0.308)	<.001	–1.32 (0.308)	<.001
	Wave = 2			–1.52 (0.333)	<.001	–1.51 (0.333)	<.001
	Age					–0.40 (0.791)	
	Education					–1.10 (0.792)	
	Variance components						
	Level 1: within-person (residual)	2.96 (0.454)	<.001	2.24 (0.345)	<.001	2.25 (0.345)	<.001
	Level 2: in intercept	6.76 (1.615)	<.001	7.03 (1.614)	<.001	6.67 (1.543)	<.001
	Goodness of fit						
	Deviance	616.80		593.42		591.23	
	AIC	622.80		603.42		605.23	
	BIC	631.47		617.87		625.46	
**Attitude workability**							
	Fixed effects	10.68 (0.409)	<.001	10.83 (0.472)	<.001	11.83 (0.707)	<.001
	Intercept (initial status)						
	Time (rate of change)						
	Wave = 1			–0.08 (0.415)		–0.08 (0.415)	
	Wave = 2			–0.47 (0.451)		–0.46 (0.452)	
	Age					–1.24 (0.791)	
	Education					–0.78 (0.792)	
	Variance components						
	Level 1: within-person (residual)	4.20 (0.642)	<.001	4.13 (0.632)	<.001	4.14 (0.635)	<.001
	Level 2: in intercept	6.50 (1.666)	<.001	6.58 (1.676)	<.001	5.98 (1.565)	<.001
	Goodness of fit						
	Deviance	652.52		651.35		647.93	
	AIC	658.52		661.35		661.93	
	BIC	667.21		675.84		682.21	
**Attitude helping**							
	Fixed effects	8.07 (0.241)	<.001	8.17 (0.315)	<.001	8.00 (0.452)	<.001
	Intercept (initial status)						
	Time (rate of change)						
	Wave = 1			1.16 (0.587)		–0.51 (0.365)	
	Wave = 2			0.31 (0.484)		0.31 (0.392)	
	Age					0.38 (0.479)	
	Education					–0.04 (0.479)	
	Variance components						
	Level 1: within-person (residual)	3.32 (0.507)	<.001	3.17 (0.484)	<.001	3.16 (0.482)	<.001
	Level 2: in intercept	1.58 (0.594)	.008	1.61 (0.587)	.006	1.59 (0.580)	.006
	Goodness of fit						
	Deviance	577.25		572.78		572.15	
	AIC	583.25		582.78		586.15	
	BIC	591.92		597.24		606.39	
**Attitude responsibility**							
	Fixed effects						
	Intercept (initial status)	4.68 (0.248)	<.001	5.08 (0.274)	<.001	4.99 (0.428)	<.001
	Time (rate of change)						
	Wave = 1			–0.62 (0.208)	.004	–0.61 (0.208)	.004
	Wave = 2			–0.69 (0.225)	.003	–0.68 (0.225)	.003
	Age					0.54 (0.489)	
	Education					–0.37 (0.490)	
	Variance components						
	Level 1: within-person (residual)	1.18 (0.181)	<.001	1.02 (0.157)	<.001	1.02 (0.157)	<.001
	Level 2: in intercept	2.52 (0.611)	<.001	2.58 (0.612)	<.001	2.49 (0.591)	<.001
	Goodness of fit						
	Deviance	491.42		479.80		478.11	
	AIC	497.42		489.80		492.11	
	BIC	506.09		504.25		512.34	

^a^AIC: Akaike information criterion.

^b^BIC: Bayesian information criterion.

## Discussion

### Principal Results

In this study we targeted the development and pilot evaluation of a digital game-based training program for managers to promote employee mental health and reduce mental illness stigma at work. Our study contributes to strengthen the evidence base that interventions targeting leaders may be effective in improving mental health literacy and reducing mental illness stigma in the workplace. In line with prior research and our hypotheses, we found statistically significant improvements in managers’ knowledge of mental health and mental illness, attitudes toward people with mental health problems, and self-efficacy to deal with mental health situations at work, with the exception of intentions to promote employee mental health [50-52]. While these results can only be considered preliminary until replicated in a controlled trial, they nevertheless highlight some interesting findings that will help inform, first, the future development of effective antistigma interventions in the workplace and, second, relevant stakeholders such as personnel in human resources or health management about the benefits of investing in stigma reduction efforts.

Knowledge of mental health and mental illness is a key stigma component and a common target of antistigma interventions, as it enables recognition and is thus essential to the prevention of mental health problems [47]. In line with previous studies [53,54], we found improvements in managers’ knowledge of mental health and mental illness (MAKS and quiz). Research shows that improved knowledge of mental health problems strongly influences a person’s ability not only to recognize signs of mental illness, but also to seek help and support others in seeking help, and to accept treatment [55].

Evidence of the potential impact of workplace antistigma interventions on managers’ attitudes toward people with mental health problems is generally mixed [17]. While some studies did not find any significant change in overall attitudes toward people with mental health problems [53,54], others reported improvements [56,57]. In our study, we evaluated not only overall attitude but also specific aspects of attitude, namely avoidance, perceived dangerousness, beliefs about workability and competencies, helping, and responsibility. While we found decreasing overall stigmatizing attitudes in managers over time, this did not apply to attitudes related to beliefs about workability and competency of people with mental health problems, nor to attitudes related to helping. An important finding of our study is therefore that a more thorough evaluation of attitudes considering specific themes, such as perceived dangerousness or social avoidance, is necessary and may be crucial to a better understanding of the effectiveness of antistigma interventions.

Behavioral change is key to creating an open and supportive work environment [58]. While public health efforts have often failed to change behavior, antistigma interventions in the workplace were suggested to be particularly promising because they allow for clear instructions with regard to how one is expected to behave in specific situations at work [21]. In line with prior studies, we found LMHP to have a positive impact on managers’ self-efficacy to deal with mental health situations at work (eg, provide support) [51,59]. This is very important, since, even more so than knowledge, the level of self-efficacy strongly influences whether a person will engage in learned behaviors [42,60].

An open question is why LMHP did not lead to improvements in attitudes related to beliefs about workability and competency of people with mental health problems, and in managers’ intentions to promote employee mental health. One potential reason might be that managers in our sample already had quite positive attitudes at baseline regarding workability and competency of people with mental health problems, as well as intentions to promote employee mental health, which left little room for improvement postintervention. Moreover, even though people with mental health problems can function productively at work, the literature shows that employers’ beliefs about the workability and competency of people with mental health problems are often poor and may be particularly hard to change [61]. Somewhat surprisingly, attitudes related to helping employees with mental health problems if they, for example, got behind in their work were and remained relatively negative despite the training. This could be related to managers’ concerns about the equity of the distribution of responsibilities and meeting productivity pressures [62]. Having in mind how important these outcomes are to reduce stigma and given that many people with mental health problems are either unemployed but want to work or are working [63,64], we recognize that LMHP and other future workplace antistigma interventions might need to incorporate modules that address those aspects more specifically.

Due to a lack of sufficient follow-up in relevant prior studies, conclusions regarding the effectiveness of workplace antistigma interventions over the long term are limited [17]. However, the few studies that conducted a follow-up reported that changes achieved in people’s knowledge, attitudes, and behavior were, in part, sustained over time [30,53,54,56,65,66]. We also found that effects of LMHP on managers’ knowledge, attitudes, and self-efficacy were largely sustained over a 3-month period (Table 5 and Table 6). While still being significantly different from baseline values, scores seemed to slightly decrease again from post- to follow-up assessment, indicating a potential need for booster sessions and further measures.

While the use of digital game-based interventions in mental health promotion is scarce and especially so in the workplace, research in other settings such as schools shows promising effects, including significant improvements in students’ psychological well-being and increased engagement in a learning program [27,28,67]. While existing efforts, however, mainly focus on risk prevention [67,68], LMHP trained managers equally in how they can contribute to reducing symptoms of mental illness in employees and in how to enhance their psychological well-being. Digital mental health promotion interventions need to shift their traditional focus on treatment and risk prevention of mental health problems to emphasizing positive psychology, healthy leadership, and the strengthening of individual resources in healthy people in order to be of greater relevance and applicability for organizations. Compared with other nongamified workplace mental health interventions with often low participant rates [27,66], this study confirmed the growing evidence that digital game-based interventions may increase user engagement and learning attainment, thus making it an attractive strategy to facilitate widespread behavioral and cultural change in organizations [34].

### Strengths and Limitations

This pilot study contributes to strengthen the evidence base of (digital) workplace antistigma interventions. Previous efforts in mental health promotion have largely neglected the role of leaders and instead have focused on employee-level interventions to address stress at work [7,10]. A marked strength of this study is therefore its focus on managers. Additionally, it addressed (1) a lack of research in private sector organizations, (2) a lack of interventions targeting all 3 dimensions of stigma, and (3) a lack of long-term follow-up that characterizes the available literature. Furthermore, this study could help explain prior mixed findings on attitudinal change by investigating the impact of LMHP on attitudes related to specific themes rather than on a single attitude scale [17]. To the best of our knowledge, LMHP is the first digital game-based training for managers aiming to promote employee mental health and reduce mental illness stigma at work. Thus, this pilot adds to the small pool of digital workplace mental health promotion and antistigma interventions [33], providing further evidence suggesting, first, that brief Web-based interventions can be as effective as more time-consuming face-to-face equivalents, which often do not match business demands [22], and second, that incorporating gamification into the learning strategy can increase participant engagement [34].

This pilot study has some limitations that must be mentioned. First, the study lacked a control group due to formal restrictions of the participating site. To what extent observed changes were due to the intervention is therefore questionable. To account for that, we recorded whether managers participated in further interventions during the study time, and the majority did not (30/48, 63%). Second, to measure knowledge, we developed our own quiz, which was not validated. Therefore, we used a second standardized instrument (MAKS, see Methods) and found similar change patterns in knowledge over time with both instruments. Third, while the OMS-WA as an adapted version of the OMS-HC [25] has been used extensively in program evaluations [66], an evaluation of the psychometric properties of this measure has yet to be published. However, a validation study of OMS-WA is under review. Fourth, the intervention was carried out in the United Kingdom and, thus, participants might have been presensitized as a result of increased stigma reduction efforts that have been going on in the United Kingdom in the past decade [31,69-71]. This might explain the good baseline values and small changes over time and ultimately may have led to an underestimation of the real training impact. Future evaluations should aim to investigate the effectiveness of LMHP in countries where mental illness stigma might be particularly strong and prevailing and where evidence about the effectiveness of antistigma interventions is scarce [72]. Fifth, we collected no data from employees on mental health, intentions to seek help, and perceived management support, nor on actual help-seeking in this study. However, in this pilot, we specifically wanted to gain first evidence on the effectiveness of LMHP before investigating any potential indirect effects on employees. Sixth, we collected no information on user satisfaction with the digital game-based training that would allow us to make objective inferences about acceptance of and engagement with the training. However, some pretests were done to rule out any technical obstacles that could possibly undermine user satisfaction, and the digital game-based training solution was developed based on suggestions made by employees of the participating organization during semistructured interviews upfront. Furthermore, we received a vast amount of positive feedback on LMHP unofficially on completion of the pilot trial, which seems to be mirrored in the high participation rate of 89% (48/54).

### Implications for Future Research

Future analysis of data on employees and on EAP utilization, sickness absence rates, or the frequency and duration of disability claims before and after using the training program is essential in evaluating the full impact of LMHP. As the ultimate goal of the training was to create an inclusive and supportive working culture where employees feel comfortable to talk about mental health openly and seek help (early), it would be valuable to include employees’ perceptions on whether they feel supported by leaders, and whether and how that changed after the training. Investigating a change in objective data related to employee help-seeking would help establish the business case of investing in antistigma interventions in the workplace.

Even though we cannot be certain, it is very unlikely that a single intervention may be sufficient to end mental illness stigma and change the working culture in an organization. Hence, future research should explore whether training managers is an effective means of supporting employees with mental health problems or whether other interventions targeting employees instead or dual approaches (eg, campaign and training) may be more efficient to achieve cultural change in the long term. Finally, to increase the generalizability of our findings, workplace antistigma interventions targeting employees of different hierarchies in different types of workplaces are needed. Another appealing contribution of future research would be to compare different training formats (game-based vs standard Web-based vs face-to-face) and their effect on user engagement and learning attainment. In general, more digital workplace mental health interventions are needed that incorporate elements of positive psychology and focus on keeping employees healthy, motivated, and productive.

### Conclusions

This pilot study provides first evidence on the effectiveness of LMHP, demonstrating its ability to positively affect managers’ knowledge, attitudes, and self-efficacy to deal with mental health situations at work. Further evaluation is needed to investigate potential beneficial effects on employees’ perceptions of management support, on their acceptance and use of existing mental health interventions (eg, EAP), and on the working culture in an organization. The benefits of digital game-based learning, such as increased participant engagement and reach, make it an effective strategy to facilitate widespread behavioral and cultural change in organizations.

## Abbreviations

EAP: employee assistance program
LMHP: Leadership Training in Mental Health Promotion
LMU: Ludwig-Maximilians-University Munich
MAKS: Mental Health Knowledge Schedule
OMS-HC: Opening Minds Scale for Health Care Providers
OMS-WA: Opening Minds Scale on Workplace Attitudes
